# *JAK2* p.G571S in B-cell precursor acute lymphoblastic leukemia: a synergizing germline susceptibility

**DOI:** 10.1038/s41375-019-0459-z

**Published:** 2019-04-09

**Authors:** Minhui Lin, Karin Nebral, Christoph G. W. Gertzen, Ithamar Ganmore, Oskar A. Haas, Sanil Bhatia, Ute Fischer, Michaela Kuhlen, Holger Gohlke, Shai Izraeli, Jan Trka, Jianda Hu, Arndt Borkhardt, Julia Hauer, Franziska Auer

**Affiliations:** 1 Department of Pediatric Oncology, Hematology and Clinical Immunology, Heinrich-Heine University Düsseldorf, Medical Faculty, Düsseldorf, Germany; 2 Fujian Institute of Hematology, Fujian Provincial Key Laboratory of Hematology, Fujian Medical University Union Hospital, 350001 Fuzhou Fujian, China; 3 Childrens Cancer Research Institute, St. Anna Childrens Hospital, Vienna, Austria; 4 Institute of Pharmaceutical and Medicinal Chemistry, Heinrich-Heine-Universität Düsseldorf, 40225 Düsseldorf, Germany; 5 John von Neumann Institute for Computing (NIC), Jülich Supercomputing Centre (JSC) & Institute for Complex Systems - Structural Biochemistry (ICS 6), Forschungszentrum Jülich GmbH, 52425 Jülich, Germany; 6 Department of Neurology, Sheba Medical Center, Tel Hashomer, Israel; 7 The Joseph Sagol Neuroscience Center, Sheba Medical Center, Tel Hashomer, Israel; 8 Sackler Faculty of Medicine, Tel Aviv University, Tel Aviv, Israel; 9 Division of Pediatric Hematology and Oncology Schneider Children’s Medical Center and Tel Aviv University, Tel Aviv, Israel; 10 Pediatric Hematology/Oncology, Childhood Leukemia Investigation Prague, Prague, Czech Republic; 11 Department of Pediatrics, Pediatric Hematology and Oncology, University Hospital Carl Gustav Carus, Technische Universität Dresden, Fetscherstrasse 74, 01307 Dresden, Germany; 12 National Center for Tumor Diseases (NCT), Dresden, Germany: German Cancer Research Center (DKFZ), Heidelberg, Germany; Faculty of Medicine and University Hospital Carl Gustav Carus, Technische Universität Dresden, Dresden, Germany; Helmholtz-Zentrum Dresden - Rossendorf (HZDR), Dresden, Germany; 13 Department of Systems Biology, Beckman Research Institute and City of Hope Comprehensive Cancer Center, Pasadena, CA USA

**Keywords:** Cancer genetics, Acute lymphocytic leukaemia, B cells

## To the Editor:

Germline predispositions are involved in the development of 5% of childhood leukemias [1], although their contribution is believed to be higher. However, to reveal the full spectrum of pathogenic germline variants, individualized genomic patient analyses, in the context of the respective familial background (trio-calling), are needed.

Here, we present the finding of a germline predisposition to B-cell precursor acute lymphoblastic leukemia (BCP-ALL) that is exerted through two synergizing, separately transmitted germline variants (*JAK2* p.G571S and *STAT3* p.K370R). We demonstrate a modest proliferation potential of *JAK2* p.G571S, which is additionally increased by *STAT3* p.K370R through rewiring of intracellular signaling pathways and show that *JAK2* G571S can rescue a *STAT3* p.K370R-induced cell cycle arrest. Protein modeling of both variants structurally underlines the observed phenotypes. Furthermore, *JAK2* p.G571S could be identified in a second patient with Down syndrome ALL, emphasizing its significance as a recurrent synergizing germline susceptibility variant.

Taken together, we describe *JAK2* p.G571S as a novel germline predisposition in BCP-ALL. Moreover, our data emphasize the synergistic interplay between separately transmitted germline risk variants that can render B-cell precursors susceptible to additional somatic hits, allowing BCP-ALL development. Expanding this knowledge is a crucial step towards targeted treatments as well as precision-prevention programs.

Acute lymphoblastic leukemia (ALL) is the most common pediatric cancer (4/100,000) under the age of 15, with the majority of cases affecting B-cell precursors (BCP-ALL) [2]. Although survival rates exceed 90%, it remains a significant cause of death in young children. Thus, to develop novel individualized therapeutic approaches, or even better, to envision precision-prevention programs—in particularly for subtypes of high-risk ALL, elucidation of the tumor genetics are fundamental [3]. In this regard, inherited germline variants are of special interest. Recently, novel germline predisposition syndromes have been described [4], and current studies increasingly highlight the importance of trio-calling analyses in childhood cancer [5, 6].

In this study, we performed trio-calling and describe a novel scenario for lymphoid malignancies, in which two susceptibility loci are inherited—a paternal one and a maternal one—and both act synergistically in the same signaling pathway, thereby forming a susceptible B-cell precursor compartment that is prone to secondary mutations driving BCP-ALL.

**Fig. 1:**
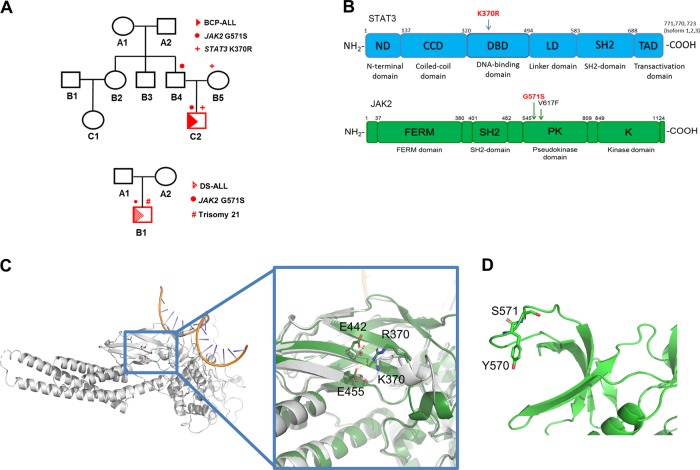
**a** Pedigree of two independent families harboring *JAK2* p.G571S. The ALL patients are marked with a red triangle, while the transmission of the identified target mutations is highlighted with red symbols (*JAK2* = circle; *STAT3* = +; trisomy 21 = #). **b** Domain organization of JAK2 and STAT3 proteins. Identified mutations (red) and the known activating *JAK2* mutation p.V617F (black), which was used as a control, are highlighted. **c** Overview of the STAT3-WT (gray) with bound DNA (orange). The blue rectangle indicates the region with the residues of interest (sticks). A close-up depicts the mutated region in STAT3-WT (gray) and the p.K370R variant (dark green). While in the STAT3-WT, p.K370 only interacts with p.E455, p.R370 of the p.K370R variant interacts with both p.E442 and p.E455 as indicated by the yellow lines. The mutation of lysine to arginine also distorts the local backbone conformation. **d** Homology model of the p.G571S variant in the JAK2 protein (green). P.S571 (sticks) is located in a loop between two β-strands next to p.Y570 (sticks), which is one of the most important phosphorylation sites in JAK2

Utilizing whole-exome sequencing we identified two concomitant germline single-nucleotide variants (SNVs) affecting the JAK2/STAT3 pathway in a boy with BCP-ALL (Fig. S1A). The *JAK2* variant, rs139504737 (c.1711G > A), leads to an aminoacid substitution from glycine to serine (p.G571S) and is rarely found within the general population (minor allele frequency (MAF) < 0.01). Interestingly, the same *JAK2* p.G571S germline variant was identified in a Down syndrome (DS-ALL) patient from an independent family (Fig. 1a). The second variant found in the BCP-ALL patient, constitutes an extremely rare, and so far for leukemia undescribed, missense mutation in the *STAT3* gene (c.1109 A > G), causing an exchange of lysine to arginine (p.K370R). While *JAK2* p.G571S was transmitted from the father, *STAT3* p.K370R was inherited from the mother’s side (Fig. 1a). Both variants are located in functionally relevant domains, with *STAT3* p.K370R being localized in the DNA-binding domain and *JAK2* p.G571S in the pseudo-kinase domain, respectively (Fig. 1b).

Furthermore, 11 somatic mutations were identified in the BCP-ALL (Fig. S1B), with a missense mutation in the *CEP89* gene (p.K138T) being the only tumor specific somatic variant. In addition, CytoScan^™^-HD analysis revealed loss of *CDKN2A* and *IKZF1* loci, which groups the patient into the recently described IKZF1^plus^ subgroup that was shown to have a particularly poor outcome [7] (Fig. S1C). Moreover, a somatic *JAK2* rearrangement was detected by cytogenetic analysis in the second *JAK2* allele. While molecular genetic analysis could rule out a classical *JAK2* fusion with *ETV6*, *BCR*, or *PAX5*, the actual fusion partner was not identified. The patient was enrolled into the AIEOP-BFM 2009 therapy protocol, responded poorly to therapy (prednisone-poor response at day 8, nonremission at day 33) and underwent hematopoietic stem cell transplantation from a matched unrelated donor after achieving first remission (MRD level < 10^−4^).

To understand potential phenotypic influences of both mutant proteins on a structural level, we generated homology models of the mutated pseudokinase domain of JAK2 and the mutated STAT3, and compared the models to their respective wild types.

*STAT3* p.K370R is located in a loop adjacent to the DNA binding site (Fig. 1c). Moreover, *STAT3* p.K370 is an important site for acetylation, which enables STAT3’s interaction with RELA, in turn promoting further downstream signaling [8]. In the deacetylated state, STAT3 p.K370 interacts with p.E455 in a β-sheet. Substitution of lysine to arginine at position p.370 has two implications: first, the arginine can interact with both p.E455 and p.E442 simultaneously, that way strengthening the interaction in the β-sheet. Second, in contrast to lysine, arginine cannot be acetylated. This combination leads to a constitutively non-acetylated form of STAT3 at position p.370, which was shown to have functional consequences in its interaction capacity with various signaling partners in HEK293T cells [8]. Furthermore, the STAT3 p.K370R protein did not show an activating phenotype in STAT3 reporter luciferase assays in HEK293T cells (Fig. S2A). This is in line with the structural modeling, suggesting impaired acetylation rather than phosphorylation (Fig. S2B).

*JAK2* p.G571 is located in a 12-residue loop connecting 2 β-strands (Fig. 1d), adjacent to p.Y570, one of the most important phosphorylation sites in JAK2. This suggests an influence on the phosphorylation of p.Y570 either through steric hindrance, interactions of the serine sidechain or changes in the backbone conformation near p.Y570. Its unique position affecting amino acid 571, which lies adjacent to the p.Y570 residue that downregulates kinase activity via autophosphorylation hints at a potential functional mechanism of p.G571S by inhibiting p.Y570-directed negative feedback.

**Fig. 2:**
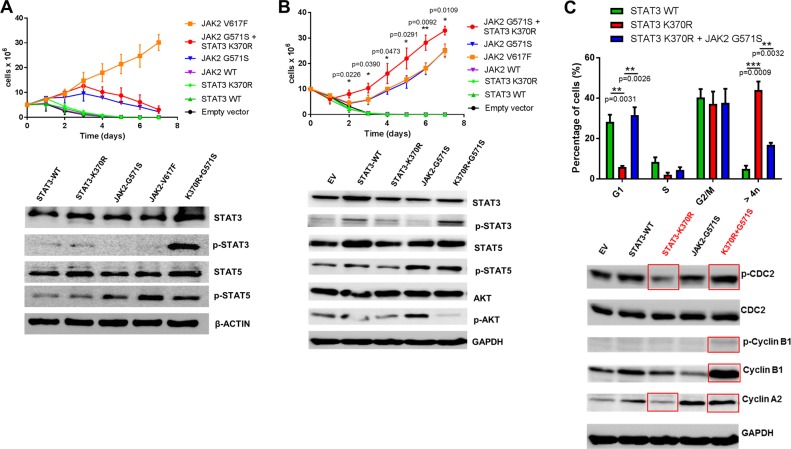
**a** Top: Proliferation curve showing prolonged survival for BaF3 cells expressing either JAK2 p.G571S protein alone or both JAK2 p.G571S and STAT3 p.K370R proteins. Transfected BaF3 cells were cultured in media without IL-3 for 7 days and their proliferation measured every day using trypan blue. JAK2 p.V617F serves as positive control. Values represent the mean out of three replicates with essentially identical datasets (*n* = 3). Bottom: Immunoblot analysis of BaF3 cells expressing STAT3-WT, STAT3-p.K370R, JAK2-p.G571S, JAK2-p.V617F, or STAT3-p.K370R+JAK2-p.G571S. Depicted are p-STAT3 and p-STAT5 levels of the respective cells. Total STAT3, STAT5, as well as beta-ACTIN serve as loading controls (*n* = 2). **b** Top: Proliferation curve showing prolonged survival for BaF3/CRLF2-IL-7RWT cells expressing either JAK2 p.G571S alone or both JAK2 p.G571S and STAT3 p.K370R. Cells harboring both mutants show a significantly higher proliferation rate compared to cells expressing JAK2 p.G571S, starting at day 2 after IL-3 withdrawal (calculated by Student’s *t* test: *p* values are indicated). Cells were cultured as described in (**a**) (*n* = 3). Bottom: Immunoblot of BaF3/CRLF2-IL-7RWT cells harboring Empty Vector (EV), STAT3-WT, STAT3-p.K370R, JAK2-p.G571S, or STAT3-p.K370R+JAK2-p.G571S expression plasmids. Depicted are p-STAT3, p-STAT5, and p-AKT levels of the respective cells. Total STAT3, STAT5, AKT, as well as GAPDH serve as loading controls (*n* = 2). **c** Top: Cell cycle analysis showing an accumulation of >4*n*, as well as a reduction of cells in the G1-phase for STAT3 p.K370R expressing BaF3 cells, as compared to cells expressing STAT3-WT or STAT3 p.K370R+JAK2 p.G571S. *p* Values are indicated as calculated by Student’s *t* test (*n* = 3). Bottom: Immunoblot depicting the expression of the cell cycle related proteins CDC2 and Cyclin-B1/B2/A2 in BaF3 cells expressing either EV, STAT3-WT, STAT3-p.K370R, JAK2-p.G571S, or both mutant proteins. GAPDH serves as loading control

To assess the cooperative potential of both variants, BaF3 depletion assays were carried out. In normal BaF3 cells, neither STAT3 p.K370R nor JAK2 p.G571S protein expression alone were sufficient to induce IL-3 independent growth, although immunoblot analyses confirmed increased p-STAT3 levels in cells expressing both JAK2 p.G571S and STAT3 p.K370R (Fig. 2a). Since dimerization by a cytokine receptor facilitates the constitutive activation of JAK2 mutants, we further transfected BaF3 cells which expressed human CRLF2 and the IL7R alpha chain (leading to the formation of the heterodimeric receptor for thymic stromal lymphopoietin (TSLP) [9]) with the identified target variants. In BaF3/CRLF2-IL-7RWT cells, JAK2 p.G571S protein expression conferred IL-3 independent growth. Moreover, the combination of both mutant proteins (JAK2 p.G571S + STAT3 p.K370R) showed a mild but significant growth advantage starting 2 days after IL-3 withdrawal (Fig. 2b). Immunoblot analysis revealed high-p-AKT levels in cells expressing JAK2 p.G571S, which was changed to p-STAT3 through additional expression of STAT3 p.K370R. Since hyperactivation of p-AKT negatively affects precursor B-cell survival [10], the here observed signal rewiring indicates a synergistic effect of both variants by balancing out signaling strengths.

Besides the cooperating capacity of both identified germline variants, we further observed that the STAT3 p.K370R mutant protein alone changed the phenotype of the cells in culture, with an accumulation of enlarged BaF3 cells. Surprisingly, this phenotype was reversed in the double mutant cells expressing both STAT3 p.K370R and JAK2 p.G571S (Fig. S2C). Cell cycle analysis was in line with this observation, showing a significant increase in >4*n* cells (*p* = 0.0009), while those in the G-1 phase were significantly decreased (*p* = 0.0031) in STAT3 p.K370R expressing BaF3 cells compared to STAT3-WT cells (Fig. 2c). Again, this phenotype was reduced in BaF3 cells transfected with both mutants simultaneously (G1 phase *p* = 0.0026; > 4*n*, *p* = 0.0032). In line with the reversed cell cycle phenotype in the double mutant cells, immunoblot analysis of the different conditions confirmed increased p-CDC-2, p-CyclinB1/Cyclin-B1, and Cyclin-A2 in BaF3 cells expressing both JAK2 p.G571S and STAT3 p.K370R compared to STAT3 p.K370R alone (Fig. 2c). Taken together, STAT3 p.K370R conferred a cell cycle arrest in BaF3 cells which is consistent with a loss-of-function phenotype in the STAT3 reporter luciferase assay and the structural modeling.

These data suggest that the two mutations can act in concert to exert a germline susceptibility toward BCP-ALL development by the accumulation of a susceptible precursor compartment. This compartment might be prone to acquire additional secondary lesions in *IKZF1* and *CDKN2A* or alterations of the JAK2 WT allele, which act as somatic oncogenic drivers leading to malignant growth.

Activation of the JAK-STAT pathway is known to be a key event in a variety of hematological malignancies. *JAK2* p.V617F leads to constitutively active STAT5 signaling in about 80% of patients suffering from myeloproliferative neoplasms [11], and a high frequency of somatic rearrangements or SNVs activating JAK2 are found in Ph-like ALL [12] and DS-ALL [13, 14]. However, germline *JAK2* mutations particularly in BCP-ALL are rare. Here, we identified a second patient harboring germline *JAK2* p.G571S in a DS-ALL cohort of 88 patients (Fig. 1a and Fig. S3) [14]. A major proportion of DS-ALL shows high expression of CRLF2 and somatic JAK/STAT pathway activation [15]. Although the analyzed DS-ALL patient did not express a P2RY8-CRLF2 transcript or show a *CRLF2* p.232 mutation, he may harbor an activating translocation of *CRLF2* into the *IGH* chain locus. However, due to the lack of patient material we could not test this hypothesis. Therefore, though CRLF2 activation in association with *JAK2* p.G571S and trisomy 21 seems highly likely, we cannot confirm an active CRLF2 status in this patient. Nevertheless, the germline *JAK2* p.G571S mutation in combination with trisomy 21 can be a complementary scenario of two germline variants acting in synergy to render cells susceptible to additional somatic alterations that can drive ALL development. This insight further strengthens the unique and important role of weak oncogenic germline risk variants (e.g., *JAK2* p.G571S) and how they can synergize with additional low-penetrance mutations/alterations to predispose to ALL development.

Thus, we suggest a scenario where *STAT3* p.K370R or trisomy 21 in synergy with *JAK2* p.G571S prime precursor B-cells susceptible for oncogenic transformation through the acquisition of secondary somatic hits. This can be a rational explanation why parents of affected children are healthy throughout life, whereas children who carry both germline variants develop ALL. Increasing knowledge of inherited di-/polygenic variants will be of great importance for the development of novel precision-prevention approaches in the future.

We are indebted to all members of our groups for useful discussions and for their critical reading of the manuscript. Special thanks go to Silke Furlan, Friederike Opitz and Bianca Killing. F.A. is supported by the Deutsche Forschungsgemeinschaft (DFG, AU 525/1-1). J.H. has been supported by the German Children’s Cancer Foundation (Translational Oncology Program 70112951), the German Carreras Foundation (DJCLS 02R/2016), Kinderkrebsstiftung (2016/2017) and ERA PerMed GEPARD. Support by Israel Science Foundation, ERA-NET and Science Ministry (SI). A.B. is supported by the German Consortium of Translational Cancer Research, DKTK. We are grateful to the Jülich Supercomputing Centre at the Forschungszemtrum Jülich for granting computing time on the supercomputer JURECA (NIC project ID HKF7) and to the “Zentrum für Informations-und Medientechnologie” (ZIM) at the Heinrich Heine University Düsseldorf for providing computational support to H.G. The study was performed in the framework of COST action CA16223 “LEGEND”.

## Supplementary information

Supplementary Information

Supplemenary Figures S1-S3
