# Supplementary material for: JAK2 p.G571S in B-cell precursor acute lymphoblastic leukemia: a synergizing germline susceptibility
Source: Leukemia. 2019 Apr 9;33(9):2331–5. doi: 10.1038/s41375-019-0459-z (PMC6756027; doi:10.1038/s41375-019-0459-z)
Supplement: Supplementary file 1 — Supplementary Information [file 41375_2019_459_MOESM1_ESM.docx]

**Supplementary Information Lin et al.**

**Supplementary Figure Legends:**

**Fig. S1**

(**A**) Sanger Sequencing results validating the presence of the heterozygous germline variants *JAK2* p.G571S and *STAT3* p.K370R in the BCP-ALL patient’s tumor (initial) and germline (remission) sample (Reverse strand shown).

(**B**) Whole Exome sequencing analysis depicting overall, somatic and somatic cancer (filtered according to the COSMIC cancer gene consensus list) variants found by comparing germline and tumor sample of the BCP-ALL patient.

(**C**) CytoScan™ HD analysis showing deletions of *IKZF1* exons 2-7 and *CDKN2A.* The weighted log2 ratios (copy number profiles) are depicted in the upper part, genes are indicated by black vertical bars; SNP markers, light green vertical bars; oligo markers, dark green vertical bars are indicated in the lower part of the Figure.

**Fig. S2**

(**A**) STAT3 luciferase reporter assay in HEK293T cells stably expressing STAT3 WT, p.K370R or p.K392R (positive control). The activity of STAT3 WT and the mutants could be reduced by the addition of the STAT3 inhibitor S3I-201. The empty vector control was subtracted from all values. Mean values and standard deviations are shown (n=3).

(**B**) Immunoblot analysis depicting pSTAT3 levels in HEK239T cells transfected with STAT3 constructs. Total STAT3 and β-ACTIN serve as loading controls.

(**C**) Microscopic picture of enlarged BaF3 cells expressing STAT3 p.K370R (red arrow), as compared to STAT3-WT or STAT3 p.K370R + JAK2 p.G571S expressing cells.

**Fig. S3**

(A) Family pedigree of the DS-ALL patient. Sanger Sequencing confirms the presence of the heterozygous germline variant *JAK2* p.G571S (Forward strand shown).

**Supplementary Methods**

**Cell lines**

BaF3 cells were obtained from DMSZ (ACC 300, Heidelberg), while BaF3/CRLF2-IL-7RWT cells were kindly provided by Shai Isreali (1). Both cells lines were cultivated in RPMI 1640 Medium GlutaMAX^TM^ (Life Technologies), supplemented with 10% (v/v) heat inactivated fetal calf serum, Gentamycin (50 µg/ml) and recombinant mouse IL-3 (10 µg/ml) (Life Technologies), at 37°C and 5% CO_2_. HEK293T cells were cultivated in DMEM (1x) + GlutaMAX^TM^, 10% FBS (heat inactivated), 1% Penicillin-Streptomycin (10,000 U/mL), 1% Sodium Pyruvate (100 mM), 1% MEM Non-Essential Amino Acids Solution (100X) (all supplied by Gibco) at 37°C and 5% CO_2_. All cell lines are either authenticated by the DSMZ or by collaboration partners (within the last 6 months of thawing). All cell lines tested negative for mycoplasma.

**Sequencing**

Sample acquisition: The AllPrep DNA/RNA Mini Kit (Qiagen, Hilden, Germany) was used to purify DNA according to the manufacturer’s instructions. The study was approved by the ethics committee of the Heinrich-Heine-University Düsseldorf and informed consent was obtained from all subjects.

Exome library preparation and next generation sequencing: Exome library preparation was performed using the Agilent SureSelectXT Human All Exon kit with modifications adapted from Fisher et al. (2). Briefly, we added SPRI beads to the original protocol and reduced the size of the reaction to 0.5 µl in order to be able to use PCR tubes for subsequent steps. Furthermore, we reduced the volume for washing. We minimized sample loss and optimized sample processing by reducing sample handling. We therefore just added freshly prepared 20% PEG/2.5M NaCl (Sigma) instead of elution of samples from the SPRI beads for library preparation. Targeted capture by hybridization to an RNA library was performed according to the manufacturer’s protocol. Purification and enrichment of the captured library was achieved by binding to MyOne Streptavidin T1 Dynabeads (LifeTechnolgies) and off-bead PCR amplification in the linear range. 2x100bp sequencing with a 6bp index read was performed using the TruSeq SBS Kit v3 on the HiSeq 2500 (Illumina).

Data analysis: Fastq files were generated by using BcltoFastq 1.8.4 (Illumina). BWA version 0.7.4. (3) was used to align sequence data to the human reference genome (GRCh38.p12). Conversion steps were carried out using Samtools (4, 5) followed by removal of duplicate reads (http://broadinstittute.github.io/picard). Local realignment around indels, SNP-calling, annotation and recalibration was facilitated by GATK 2.4.9 (6). Resulting variation calls were annotated by Variant Effect Predictor (7) using the Ensembl database (v70) and imported into an in-house MySQL database to facilitate automatic and manual annotation, reconciliation and data analysis by complex database queries. Loss of function prediction scores for PolyPhen2 (8) and SIFT (9) were extracted from this Ensemble release (10). Somatic calls were produced using MuTect (11) and VarScan2 (12). Further filtering was applied to consider only entries with at least 9% difference in allele frequency, between tumor and normal samples, for further analysis. Cancer related genes were determined by the cancer gene consensus from COSMIC (13).

Mutations were validated using Sanger sequencing on a 3130 Genetic Analyzer (Applied Biosystems). The following primer pairs were used:

| **Gene** | **primer sequences 5'-3'** |
| --- | --- |
| h*JAK2* | *forward*: GGC CAA GGC ACT TTT ACA AAG ATT |
|  | *reverse:* GCT GCA CAC ATG AGT ACG TT |
| *hSTAT3* | *forward*: TTG CTG GTC AAA TTC CCT GAG TTG |
|  | *reverse:* GTG GCT TTG TTC AGA CAC GTA |

**Cloning**

A cDNA encoding the CDS of wildtype human *JAK2* and *STAT3* were obtained via PCR from the vectors pDONR223-*JAK2* (14) (gift from William Hahn & David Root - Addgene plasmid #23915) and pLEGFP-WT-*STAT3* (15) (gift from George Stark - Addgene plasmid #71450), using the Phusion High-Fidelity DNA Polymerase (Thermo Scientific). The mutant cDNAs for *JAK2* p.G571S, *JAK2* p.V617F and *STAT3* p.K370R and *STAT3* p.K392R were created by site directed mutagenesis by PCR with the same polymerase. The following primer pairs were used:

| **Gene** | | **primer sequences 5'-3'** |
| --- | --- | --- |
| *JAK2* WT | *forward:* GGC GCa cgc gtg cca ccA TGG GAA TGG CCT GCC TTA CGA TG | |
|  | *reverse*: CCT CGa cta gtt cat ccA GCC ATG TTA TCC CTT ATT TGA TCC | |
| *JAK2* p.G571S | *forward*: CGA AGA GAA GTA GGA GAC TAC AGT CAA CTG CAT GAA ACA G | |
|  | *reverse*: CTG TTT CAT GCA GTT GAC TGT AGT CTC CTA CTT CTC TTC G | |
| *JAK2* p.V617F | *forward*: GGT TTT AAA TTA TGG AGT ATG TTT CTG TGG AGA CGA G | |
|  | *reverse*: CTC GTC TCC ACA GAA ACA TAC TCC ATA ATT TAA AAC C | |
| *STAT3* WT | *forward*: GGC GCa cgc gtg cca ccA TGG CCC AAT GGA ATC AGC TAC AG | |
|  | *reverse*: CCT CGa cta gtT CAC ATG GGG GAG GTA GCG CAC TCC GAG G | |
| *STAT3* p.K370R | *forward*: CTT AAA ATT AAA GTG TGC ATT GAC AgA GAC TCT GGG GAC G | |
|  | *reverse*: CGT CCC CAG AGT CTc TGT CAA TGC ACA CTT TAA TTT TAA G | |
| *STAT3* p.K392R | *forward*: CAT TCT GGG CAC AAA CAC AAg AGT GAT GAA CAT GGA AGA ATC | |
|  | *reverse*: GAT TCT TCC ATG TTC ATC ACT cTT GTG TTT GTG CCC AGA ATG | |

The wildtype and mutant *JAK2* and *STAT3* sequences were cloned into a derivative of a bicistronic expression vector (pMC3) used previously for stable expression of genes in cell lines (16). Here, the vector was additionally modified to either encode the Hygromycin or Puromycin resistence gene as the second cistron (pMC3.Hygro or pMC3.Puro respectively). The identity of the respective cDNAs was confirmed by Sanger sequencing. BaF3 cells were transfected using the Amaxa Nucleofector Technology (Lonza), according to manufacturer’s protocol. Briefly, BaF3 cells (5 x 10^6^) were resuspended in 100 µl Amaxa Nucleofector solution V containing either 5 µg of pMC3-*JAK2*.Hygro^WT/Mutant^, pMC3-*STAT3*.Hygro^WT/Mutant^ or the empty vector and electroporated using the Nucleofector program X-01. For the double mutant cells pMC3-*JAK2*.Hygro^G571S^ and pMC3-*STAT3*.Puro^K370R^ were used. Cells harboring the respective plasmids were selected using either 600 µg/ml Hygromycin B (Life Technologies) or 2 µg/ml Puromycin (Sigma).

**BaF3 Proliferation**

BaF3 cells expressing either JAK2^WT/Mutant^, STAT3^WT/Mutant^ or the empty vector control, were washed twice with medium without IL-3, before they were cultured in the absence of IL-3 for 7 days. Proliferation was measured by counting the cells every day using Trypan Blue (Sigma-Aldrich).

**Cell Cycle Analysis**

Nicoletti cell cycle assay was carried out as described previously (17). In short, 1 x 10^6^ cells were washed once with PBS, before being resuspended in 500 µl fluorochrome solution and stained over night at 4°C. The samples were analyzed by ﬂow cytometry using the 488-nm laser for excitation.

**Luciferase reporter assays**

*STAT3* constructs (WT, p.K392R, p.K370R) were transfected into HEK293T using Lipofectamin LTX (Thermo Fisher Scientific) and selected with 200 μg/ml hygromycin. Positive clones were subsequently transfected with a dual-luciferase reporter system (Cignal STAT3 Reporter, Qiagen) according to the manufacturer’s instructions. Renilla luciferase served as a control of transfection efficiency. Luciferase activity was determined in untreated cells and cells treated with 50 μM S3I-201 (STAT3 inihibitor) after 24 h using the Dual-Glo® Luciferase Assay (Promega) and a Spark 10M plate reader (Tecan Group). STAT3 activity was determined by comparing the normalized luciferase activities of the reporter in cells stably expressing mutant STAT3 compared to STAT3 WT. Assays were performed in triplicates.

**Immunoblotting**

Whole cell extracts were obtained as previously described (18) and lysed in RIPA buffer (50 mM Tris pH 8.0, 150 mM NaCl, 0,5 % Sodiumdeoxycholate, 1 % NP-40 substitute, 0,1 % SDS), containing protease and phosphatase inhibitors (Roche Diagnostics). 20 µg of whole protein was separated on SDS-PAGE and transferred to Hybond-C Extra membranes (Amersham Biosciences). Immunoblotting was carried out using the following antibodies: p-STAT3 (9145), STAT3 (9139), p-STAT5 (9359), STAT5 (25656), p-AKT (4060), AKT (4691), p-CDC2 (4539), CDC2 (9116), p-Cyclin B1 (4133), Cyclin B1 (4138), Cyclin A2 (4656), GAPDH (2118) (all from Cell Signaling) and Anti-Beta-Actin (A2228-100UL, Sigma-Aldrich). Detection was done using anti-rabbit or anti-mouse horseradish peroxidase conjugates (Santa Cruz Biotechnology), respectively, with an ECL system (Thermo Scientific).

**Homology modeling**

We modeled the STAT3 p.K370R mutant protein (UniProt-ID: P40763) using the X-ray crystal structures of STAT1 (PDB-ID: 1BF5) and STAT3 (PDB-ID: 3CWG) as templates and our in-house modeling suite TopModel (19, 20). Similarly, we modeled the p.G571S variant of the Pseudokinase domain of the JAK2 protein (UniProt-ID: O60674; residues 545-809) using the domain’s X-ray crystal structure (PDB-ID: 4BBE) as a template.

**Supplementary References**

1. Shochat C, Tal N, Bandapalli OR, Palmi C, Ganmore I, te Kronnie G, et al. Gain-of-function mutations in interleukin-7 receptor-alpha (IL7R) in childhood acute lymphoblastic leukemias. The Journal of experimental medicine. 2011;208(5):901-8.

2. Fisher S, Barry A, Abreu J, Minie B, Nolan J, Delorey TM, et al. A scalable, fully automated process for construction of sequence-ready human exome targeted capture libraries. Genome biology. 2011;12(1):R1.

3. Li H, Durbin R. Fast and accurate long-read alignment with Burrows-Wheeler transform. Bioinformatics. 2010;26(5):589-95.

4. Li H, Durbin R. Fast and accurate short read alignment with Burrows-Wheeler transform. Bioinformatics. 2009;25(14):1754-60.

5. Li H, Handsaker B, Wysoker A, Fennell T, Ruan J, Homer N, et al. The Sequence Alignment/Map format and SAMtools. Bioinformatics. 2009;25(16):2078-9.

6. DePristo MA, Banks E, Poplin R, Garimella KV, Maguire JR, Hartl C, et al. A framework for variation discovery and genotyping using next-generation DNA sequencing data. Nature genetics. 2011;43(5):491-8.

7. McLaren W, Pritchard B, Rios D, Chen Y, Flicek P, Cunningham F. Deriving the consequences of genomic variants with the Ensembl API and SNP Effect Predictor. Bioinformatics. 2010;26(16):2069-70.

8. Adzhubei IA, Schmidt S, Peshkin L, Ramensky VE, Gerasimova A, Bork P, et al. A method and server for predicting damaging missense mutations. Nature methods. 2010;7(4):248-9.

9. Kumar P, Henikoff S, Ng PC. Predicting the effects of coding non-synonymous variants on protein function using the SIFT algorithm. Nature protocols. 2009;4(7):1073-81.

10. Boeva V, Popova T, Bleakley K, Chiche P, Cappo J, Schleiermacher G, et al. Control-FREEC: a tool for assessing copy number and allelic content using next-generation sequencing data. Bioinformatics. 2012;28(3):423-5.

11. Cibulskis K, Lawrence MS, Carter SL, Sivachenko A, Jaffe D, Sougnez C, et al. Sensitive detection of somatic point mutations in impure and heterogeneous cancer samples. Nature biotechnology. 2013;31(3):213-9.

12. Koboldt DC, Zhang Q, Larson DE, Shen D, McLellan MD, Lin L, et al. VarScan 2: somatic mutation and copy number alteration discovery in cancer by exome sequencing. Genome research. 2012;22(3):568-76.

13. Futreal PA, Coin L, Marshall M, Down T, Hubbard T, Wooster R, et al. A census of human cancer genes. Nat Rev Cancer. 2004;4(3):177-83.

14. Johannessen CM, Boehm JS, Kim SY, Thomas SR, Wardwell L, Johnson LA, et al. COT drives resistance to RAF inhibition through MAP kinase pathway reactivation. Nature. 2010;468(7326):968-72.

15. Dasgupta M, Unal H, Willard B, Yang J, Karnik SS, Stark GR. Critical role for lysine 685 in gene expression mediated by transcription factor unphosphorylated STAT3. The Journal of biological chemistry. 2014;289(44):30763-71.

16. Linka RM, Risse SL, Bienemann K, Werner M, Linka Y, Krux F, et al. Loss-of-function mutations within the IL-2 inducible kinase ITK in patients with EBV-associated lymphoproliferative diseases. Leukemia. 2012;26(5):963-71.

17. Riccardi C, Nicoletti I. Analysis of apoptosis by propidium iodide staining and flow cytometry. Nature protocols. 2006;1(3):1458-61.

18. Martin-Lorenzo A, Hauer J, Vicente-Duenas C, Auer F, Gonzalez-Herrero I, Garcia-Ramirez I, et al. Infection Exposure is a Causal Factor in B-cell Precursor Acute Lymphoblastic Leukemia as a Result of Pax5-Inherited Susceptibility. Cancer discovery. 2015;5(12):1328-43.

19. Widderich N, Pittelkow M, Hoppner A, Mulnaes D, Buckel W, Gohlke H, et al. Molecular dynamics simulations and structure-guided mutagenesis provide insight into the architecture of the catalytic core of the ectoine hydroxylase. Journal of molecular biology. 2014;426(3):586-600.

20. Zhang Z, Gu Q, Jaguva Vasudevan AA, Hain A, Kloke BP, Hasheminasab S, et al. Determinants of FIV and HIV Vif sensitivity of feline APOBEC3 restriction factors. Retrovirology. 2016;13(1):46.
