# Supplementary material for: JAK2 p.G571S in B-cell precursor acute lymphoblastic leukemia: a synergizing germline susceptibility
Source: Leukemia. 2019 Apr 9;33(9):2331–5. doi: 10.1038/s41375-019-0459-z (PMC6756027; doi:10.1038/s41375-019-0459-z)
Supplement: Supplementary file 2 — Supplemenary Figures S1-S3 [file 41375_2019_459_MOESM2_ESM.pptx]

## Slide 1
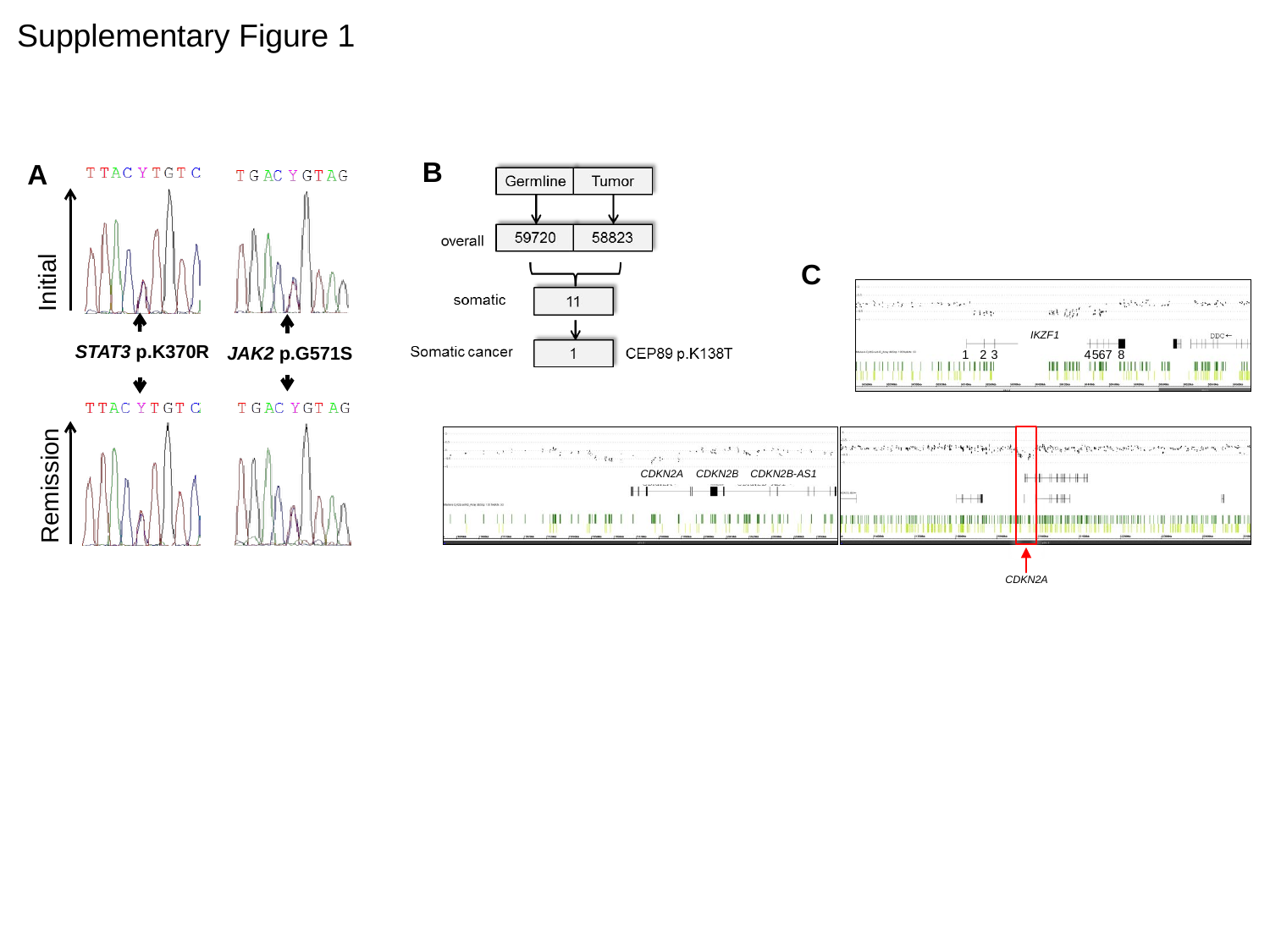

Supplementary Figure 1
B
A
Initial
STAT3 p.K370R
JAK2 p.G571S
Remission
C
1
2
3
4
5
6
7
8
IKZF1
CDKN2A
CDKN2A
CDKN2B
CDKN2B-AS1

## Slide 2
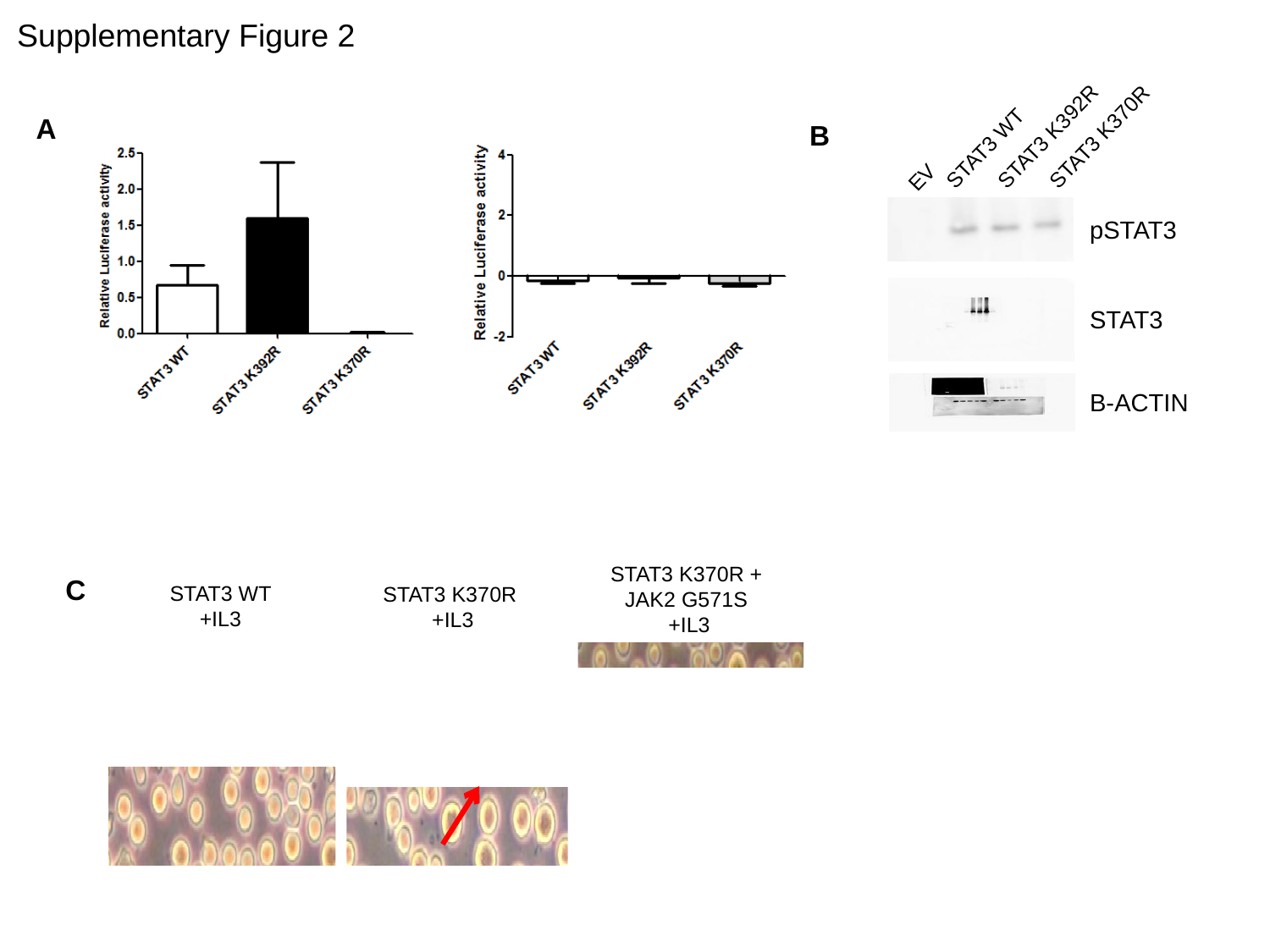

Supplementary Figure 2
STAT3 K392R
STAT3 K370R
STAT3 WT
EV
pSTAT3
STAT3
Β-ACTIN
A
B
STAT3 K370R +
JAK2 G571S
+IL3
STAT3 WT
+IL3
STAT3 K370R
+IL3
C

## Slide 3
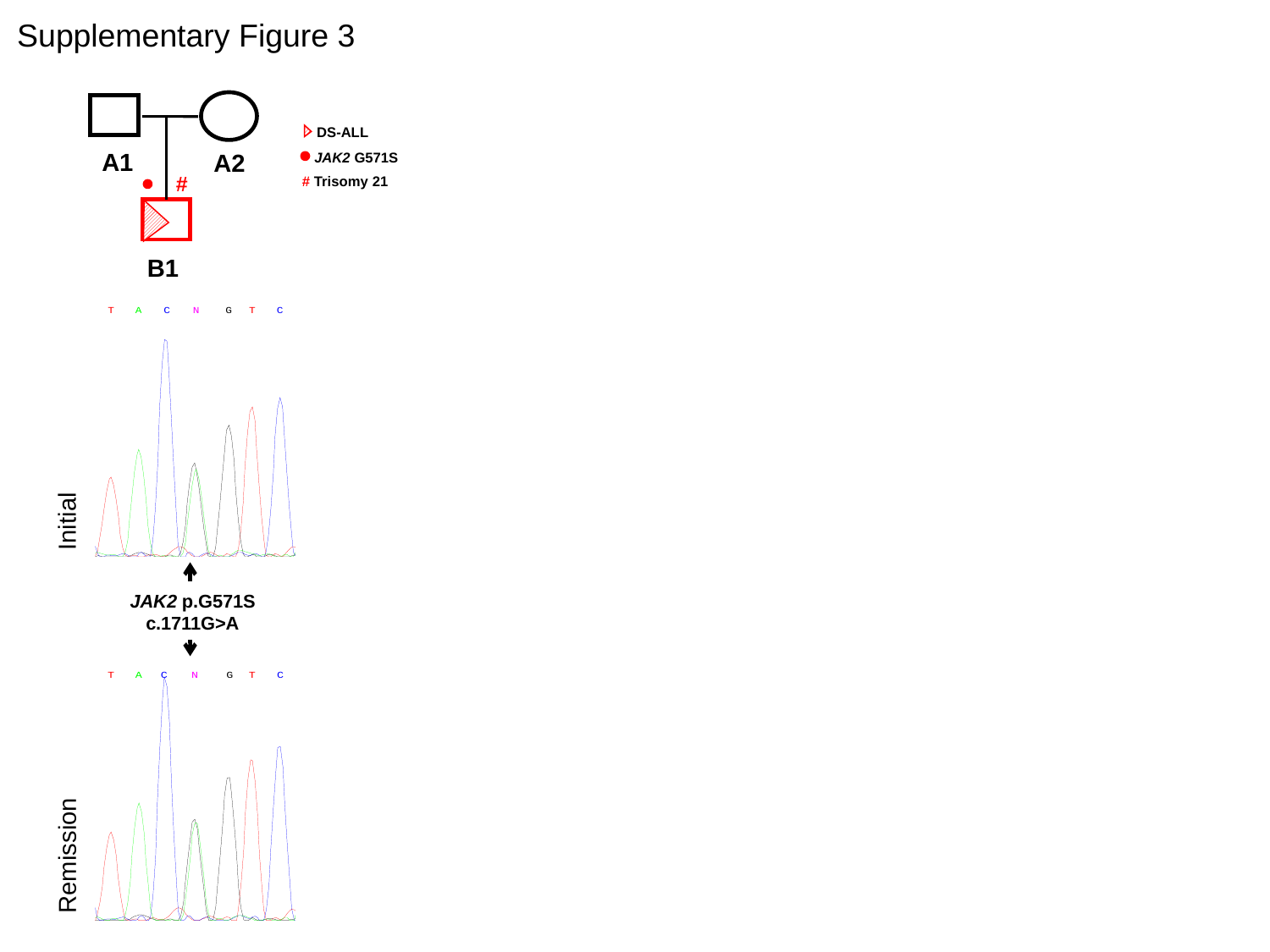

Supplementary Figure 3
A1
A2
#
B1
 DS-ALL
 JAK2 G571S
# Trisomy 21
Initial
JAK2 p.G571S
c.1711G>A
Remission
